# *Streptococcus* endopeptidases promote HPV infection in vitro

**DOI:** 10.1002/mbo3.628

**Published:** 2018-04-19

**Authors:** Sylvia I. Pavlova, Reid V. Wilkening, Michael J. Federle, Yu Lu, Joel Schwartz, Lin Tao

**Affiliations:** ^1^ Department of Oral Biology College of Dentistry University of Illinois at Chicago Chicago IL USA; ^2^ Department of Microbiology and Immunology College of Medicine University of Illinois at Chicago Chicago IL USA; ^3^ Department of Medicinal Chemistry and Pharmacognosy Center for Biomolecular Sciences College of Pharmacy University of Illinois at Chicago Chicago IL USA; ^4^ Department of Oral Medicine and Diagnostic Sciences College of Dentistry University of Illinois at Chicago Chicago IL USA

**Keywords:** cancer, furin, HPV, peptidase, *Streptococcu*s, transformation

## Abstract

Both cervical and throat cancers are associated with human papillomavirus (HPV). HPV infection requires cleavage of the minor capsid protein L2 by furin. While furin is present in the vaginal epithelium, it is absent in oral epithelial basal cells where HPV infection occurs. The objective of this study was to investigate whether common oral bacteria express furin‐like peptidases. By screening strains representing 12 oral *Streptococcus* and *Enterococcus* species, we identified that eight *Streptococcus* strains displayed high levels of furin‐like peptidase activity, with *S. gordonii* V2016 the highest. We constructed null mutations for 14 genes encoding putative endopeptidases in *S. gordonii* V2016. Results showed that three endopeptidases, PepO, PulO, and SepM, had furin‐like activities. All three mutants showed decreased natural transformation by chromosomal DNA, while the *pepO* mutant also showed reduced transformation by plasmid DNA, indicating involvement of these endopeptidases in competence development. The purified *S. gordonii* PepO protein promoted infection of epithelial 293TT cells in vitro by HPV16 pseudovirus. In conclusion, oral bacteria might promote HPV infection and contribute to HPV tissue tropism and subsequent carcinogenesis in the oral cavity and throat by providing furin‐like endopeptidases.

## 1 INTRODUCTION

Oral and oropharyngeal (throat) cancers are the sixth most common cancer world‐wide. Each year, about 650,000 cases are diagnosed and nearly 40% of them are fatal (Ferlay, Pisani, & Parkin, 2004). While cervical cancer is the most common human papillomavirus (HPV)‐associated cancer in women, oral and oropharyngeal cancers are the most common HPV‐associated cancers in men (CDC, 2012). In recent decades, the incidences of HPV‐associated oropharyngeal cancers have been increasing consistently in industrialized countries (Chaturvedi et al., 2011; Shiboski, Schmidt, & Jordan, 2005). HPV‐related cervical cancer is a predominant cause of mortality among women in African countries (De Vuyst et al., 2013).

To infect human tissues, viruses often require activation by host peptidases such as furin. Furin is a serine endopeptidase expressed in many mammalian cells, mainly localized in the trans‐Golgi network (Nakayama, 1997). It belongs to the family of subtilisin‐like proprotein convertases that process latent precursor proteins into their biologically active products, and it is a calcium‐dependent protease and can efficiently cleave precursor proteins at their proteolytic cleavage consensus site (‐RXXR↓‐) (Thomas, 2002). Proteolytic activation of viral attachment factors at the site of infection is a strategy shared among many viruses (Klenk & Garten, 1994), where many viral proproteins contain consensus sites for furin. These include the minor capsid L2 protein of HPV (Richards, Lowy, Schiller, & Day, 2006) and the envelope glycoprotein gB of cytomegalovirus (CMV) (Jean et al., 2000) and Epstein–Barr virus (EBV) (Sorem & Longnecker, 2009).

Among many HPV subtypes, HPV16 is the most oncogenic. Infection by HPV16 is initiated by binding to the receptor heparan sulfate proteoglycans (HSPGs) on exposed basement membrane. This exposes the L2 cleavage site. Following cleavage by furin, HPV16 invades the tissue (Raff et al., 2013; Richards et al., 2006; Schiller, Day, & Kines, 2010). Although HSPGs exist in nearly all tissues throughout the body (Sarrazin, Lamanna, & Esko, 2011), the expression of furin varies. In all layers of the mouse vaginal epithelium, furin expression is high (Kines, Thompson, Lowy, Schiller, & Day, 2009), while in the human oral epithelium, furin expression is low, detectable only in the upper spinous and surface layers, not in the lower basal cell layer, where HPV initiates infection (López de Cicco, Bassi, Page, & Klein‐Szanto, 2002). The absence of furin in local tissues cannot explain why HPV‐induced cancers commonly occur in the oral cavity and oropharynx. Apparently, a gap exists in current understanding of HPV infection. It is largely based on in vitro cell culture studies under germ‐free conditions (Schiller et al., 2010), experiments with nonhuman animals (Kines et al., 2009), and level of infection reported in specific populations, such as young men in the United States and women in Africa (Curado & Boyle, 2013; De Vuyst et al., 2013).

The oral cavity is not sterile. It is colonized by billions of microorganisms, collectively referred to as the oral microbiome (McLean, 2014) mostly growing as biofilm attached to the mucosal and tooth surfaces (Kolenbrander, 2000). For HPV to infect the oral tissue, the lack of host furin in epithelial basal cells might be compensated by furin‐like peptidases from the oral microbiome. In our previous publications, we reported that *Streptococcus* promotes HPV16 entry into oral keratinocytes and oral squamous cell carcinoma cells. These suggest a possible interplay for *Streptococcus* and other similar bacteria to contribute to HPV16 entry (Schwartz et al., 2012; Tao, Pavlova, Gasparovich, Jin, & Schwartz, 2015). It is, therefore, reasonable to investigate how these microorganisms could play a role in determining HPV tissue tropism, and subsequently, how they might contribute to the development of tissue‐specific neoplasia.

Several anatomic sites in the oral cavity and oropharynx, including the back of the mouth, the base of the tongue, and the tonsils, are subject to HPV infection. These sites have nonkeratinized thin mucosal lining that form crypts, which house a complex microbial population. Included are various bacteria and viruses that cause periodontitis, common cold, influenza, and cancer. Tissue tropism varies among different viruses, and bacteria found at specific locations in the body might provide necessary conditions for infection by specific viruses (Doolittle & Webster‐Cyriaque, 2014). Although a furin‐like peptidase has been reported in the eukaryotic parasite *Cryptosporidium parvum* (Wanyiri et al., 2007), in hundreds of bacterial species comprising the human microbiome (Human Microbiome Project, 2012), furin‐like peptidases have not been reported. Here, we continue our studies of interaction between *Streptococcus* and HPV16 entry capability. We report finding of three furin‐like peptidases in *S*. *gordonii*, characterizing one of them, and observation of its promotion of HPV infection in vitro.

## 2 MATERIALS AND METHODS

### 2.1 Bacterial strains, growth conditions, and plasmids

Bacterial strains and plasmids used in these studies are described in Table 1. Unless sources are indicated in the table, oral bacterial strains were isolated from the saliva of healthy volunteers. Their species were identified by 16S rRNA gene sequence. The saliva sampling study was approved by the Institutional Review Board of the University of Illinois at Chicago. All *Streptococcus* and *Entrococcus* strains were grown in Todd‐Hewitt (TH) broth supplemented with 0.5% yeast extract (THY) at 37°C without agitation in a candle jar. For growth of *E. coli* strains, the Luria‐Bertani (LB) broth or agar was used; for growth of *Lactobacillus* strains, the MRS broth or agar was used. For transformation of *S. gordonii*, heat‐inactivated horse serum (56°C for 30 min) was added into TH broth at 5% (THS). An overnight culture of *S. gordonii* strain in THS was diluted 1:40 into fresh THS. After 2 hr of incubation at 37°C, DNA was added and the bacterial culture was incubated for 1 hr and then plated onto TH agar supplemented with appropriate antibiotics (kanamycin, 250 μg/ml; erythromycin, 10 μg/ml; or tetracycline, 15 μg/ml). The plates were incubated at 37°C for 24 hr in a candle jar for selection of transformants. All chemicals and reagents unless otherwise indicated were purchased from Sigma‐Aldrich (St. Louis, Missouri).

**Table 1:** Bacterial strains and plasmids used in this study

Strain/Plasmid	Characteristics	Reference
*Streptococcus anginosus* ARB136	Wild‐type isolate	This study
*Streptococcus gordonii* V2016	Wild‐type isolate	Pavlova et al. (2013)
*Streptococcus infantis* ARB112‐2	Wild‐type isolate	This study
*Streptococcus mitis* 110‐5	Wild‐type isolate	This study
*Streptococcus mutans* ARB117‐1	Wild‐type isolate	This study
*Streptococcus oralis* 108	Wild‐type isolate	This study
*Streptococcus parasanguinis* ARB104‐2	Wild‐type isolate	This study
*Streptococcus pneumoniae* CP2000	CP1250; *Δcps*; Sm^R^	Weng, Piotrowski, & Morrison (2013)
*Streptococcus pyogenes* NZ131	Wild‐type isolate	Simon & Ferretti (1991)
*Streptococcus pyogenes* NS05‐24	Wild‐type isolate	This study
*Streptococcus salivarius* 101‐1	Wild‐type isolate	This study
*Streptococcus sanguinis* SK36	ATCC BAA‐1455	Todd Kitten
*Enterococcus faecalis* IS51.	Wild‐type isolate	This study
*Lactobacillus casei* OLB1a	Wild‐type isolate	This study
*Lactobacillus coryniformis* OLB5	Wild‐type isolates	This study
*Lactobacillus fermentum* OLB19b, 20b	Wild‐type isolates	This study
*Lactobacillus lactis* OLB12, 28, 32b, 36b, 43b	Wild‐type isolates	This study
*Lactobacillus oris* OLB9a	Wild‐type isolate	This study
*Lactobacillus plantarum* OLB14a,	Wild‐type isolate	This study
*Lactobacillus rhamnosus* OLB24a, 25a, 25b, 33	Wild‐type isolates	This study
*Lactobacillus salivarius* OLB21, 22L, 22S, 160S	Wild‐type isolates	This study
*S. gordonii* V2016 mutant		
*ΔsubAB*	Deletion of SGO_0316 & SGO_0317 with Km^R^	This study
*Δsgc*	Deletion of SGO_0566 with Km^R^	This study
*ΔsecA2*	Deletion of SGO_0974 with Km^R^	This study
*ΔsepA*	Deletion of SGO_2150 with Km^R^	This study
*pepM22*	Inactivation of SGO_0221 with pSF151, Km^R^	This study
*cppA*	Inactivation of SGO_0237 with pSF151, Km^R^	This study
*pepM26*	Inactivation of SGO_0408 with pSF151, Km^R^	This study
*sepM*	Inactivation of SGO_0652 with pSF151, Km^R^	This study
*pulO*	Inactivation of SGO_0664 with pSF151, Km^R^	This study
*pepZ1*	Inactivation of SGO_0796 with pSF151, Km^R^	This study
*sepB*	Inactivation of SGO_0847 with pSF151, Km^R^	This study
*pepSY*	Inactivation of SGO_1298 with pSF151, Km^R^	This study
*pepO*	Inactivation of SGO_1799 with pSF151, Km^R^	This study
*pepZ2*	Inactivation of SGO_2009 with pSF151, Km^R^	This study
*sepM‐pepO*	Inactivations with pVA891 and pSF151, Em^R^, Km^R^,	This study
*sepM‐pulO‐pepO*	Inactivations with pVA891, pSF152 and pSF151, Em^R^, Sp^R^, Km^R^	This study
*S. sanguinis* SK36 mutant		
*pepO*	Transformed with the *S. gordonii pepO* DNA, Km^R^	This study
*pulO*	Transformed with the *S. gordonii pulO* DNA, Km^R^	This study
*Escherichia coli* strain JM109	Cloning strain	Yanisch‐Perron, Vieira, & Messing, (1985)
*E. coli* DH5α	Cloning strain	Hanahan (1983)
*E. coli* BL21(DE3)	Protein overexpression strain	New England BioLabs
Plasmid		
pSF151	Streptococcal integration plasmid, 3.5 kb, Km^R^	Tao (1998)
pSF152	Streptococcal integration plasmid, 3.2 kb, Sp^R^	Tao (1998)
pVA891	Streptococcal integration plasmid, 5.9 kb, Em^R^	Tao (1998)
pA13	Lactic acid bacteria‐*E. coli* shuttle plasmid, 4.6 kb, Em^R^	Kojic et al. (2010)
pET21a	Cloning/expression, 5.4 kb, Amp^R^	Novagen
pET21a‐*pepO*	pET21a containing *S gordonii pepO*	This study

### 2.2 Furin‐like peptidase assay

Since certain viruses require furin activation in order to infect, we analyzed oral and throat bacterial strains for furin‐like peptidase activity. Bacteria were grown to mid‐exponential phase to OD_600_ of ~0.7 in THY broth for *Streptococcus* or *Enterococcus* and MRS broth for *Lactobacillus*, and 100 μl culture was harvested, washed, and resuspended in Na/MES buffer for analysis of furin‐like peptidase activity. The fluorogenic furin substrate Boc‐RVRR‐AMC (Enzo Life Sciences) at 50 μmol/L in Na/MES buffer [pH 7.0, 20 mmol/L MES [2‐(N‐morpholino) ethanesulfonic acid], 1 mmol/L CaCl_2_, and 0.01% Triton X‐100] was added. The mixture was incubated for 30 min at 37°C. The cells were removed by centrifugation. The fluorescence released from the substrate in the supernatant was read with the VICTOR X5 Plate Reader of PerkinElmer (Excitation 360 nm; Emission 460 nm). For analysis of purified peptidases, the protein was added to the reaction mixture and incubated for 30 min at 37°C before fluorescence reading. The purified recombinant human furin from New England BioLabs (NEB) was used as control.

### 2.3 Construction of mutant strains

The V2016 *sgc*,* subAB, sepA,* and *secA2* deletion mutants were obtained by allelic exchange (Pavlova, Jin, Gasparovich, & Tao, 2013), while the remaining 10 genes were inactivated by using the streptococcal integration plasmid pSF151 (Tao, 1998). Therefore, a total of 13 genetic loci were inactivated by allelic replacement or insertion duplication. *Streptococcus sanguinis* SK36 *pepO* mutant was also obtained by transforming SK36 with chromosomal DNA isolated from the *S. gordonii* V2016 *pepO* mutant. Standard recombinant DNA techniques were employed as described (Sambrook, Fritsch, & Maniatis, 1989). Multiple pairs of oligonucleotides (Integrated DNA Technologies) used in this study are shown in Table 2. Chromosomal DNA was prepared by the glass bead method (Ranhand, 1974). PCR products were purified using the QIAquick PCR Purification Kit (Qiagen, Valencia, California). DNA restriction enzymes were used under the conditions specified by the manufacturer (New England BioLabs, Ipswich, Massachusetts). Each mutant was confirmed genotypically by PCR. Due to insertion of the antibiotic resistance cassette, the size of the PCR DNA fragment became larger in the mutant than that in the wild type with primers flanking the insertion site (not shown).

**Table 2:** Oligonucleotides used in this study

Oligonucleotide (5′→3′)	Sequence	Restriction enzyme
Construction of *ΔsubAB* (SGO_0316‐0317)		
SubA‐F1	ataaggttaactcccttcgacaagctggtg	
SubA‐R1	tgatttcccggatccttgactgg	*Bam*HI
SubB‐F2	ggttctacagaattccctgatgg	*Eco*RI
SubB‐R2	actgaccaaatgctggttactcttagcatc	
Construction of *Δsgc* (SGO_0566)		
Sgc‐F1	cgtaggatcccttcgtgaccaaggat	
Sgc‐R1	tcgagaattcagtttgaggaaccttgatgat	*Eco*RI
Sgc‐F2	caggacggatccgagtttccatg	*Bam*HI
Sgc‐R2	gccttagcttgtgcataggcctctaag	
Construction of *ΔsecA2* (SGO_0974)		
secA2‐F1	ttgccagaagcctatgctg	
secA2‐R1	gctagaattcttatggaggccatggcacg	*Eco*RI
secA2‐F2	atgcggatccgcagagattatcctgatcgg	*Bam*HI
secA2‐R2	ttaagaatagccaggcgctgg	
Construction of *ΔsepA* (SGO_2150)		
SepA‐F1	gcgaccgttcgcttagaaggcgaatgctct	
SepA‐R1	gctaggatccttcgctagctacctgatcat	*Bam*HI
SepA‐F2	gaccagcattaggaattcaaatg	*Eco*RI
SepA‐R2	ttagagagactaatctttacttcgactccc	
Construction of SGO_0221 mutant		
Sgo‐221F	ctgagaattgcggtggcgac	*Tas*I
Sgo‐221R	gagccaattttcctcggctt	*Tas*I
Construction of SGO_0237 mutant		
Sgo‐237F	ttgggagatcagacaaagag	*Sau*3AI
Sgo‐237R	gcatgatcgccaaaaggttgataat	*Sau*3AI
Construction of SGO_0408 mutant		
Sgo‐408F	gcagctgtggatcctgcagt	*Bam*HI
Sgo‐408R	cagctgacctttgaattcac	*Eco*RI
Construction of SGO_0652 mutant		
Sgo‐652F	gactggtggatcatcggacc	*Sau*3AI
Sgo‐652R	cgtaaggttggatctgcaag	*Sau*3AI
Construction of SGO_0664 mutant		
Sgo‐664F	gaatgagaatttccgtcaga	*Tas*I
Sgo‐664R	cacttggtacaatttctatc	*Tas*I
Construction of SGO_0796 mutant		
Sgo‐796F	tttagatcaacaaggtattc	*Sau*3AI
Sgo‐796R	gaaagaacctgcagactgca	*Pst*I
Construction of SGO_0847 mutant		
Sgo‐847F	ggaatggaaattactactat	*Tas*I
Sgo‐847R	ttggcaatttactataatct	*Tas*I
Construction of SGO_1298 mutant		
Sgo‐1298F	gacttcccaaattggccggt	*Tas*I
Sgo‐1298R	gctcgttctaattccattga	*Tas*I
Construction of SGO_1799 mutant		
Sgo‐1799F	gtgaagaattcctcagcaga	*Eco*RI
Sgo‐1799R	gcggctatcctgcaggcgcc	*Pst*I
Construction of SGO_2009 mutant		
Sgo‐2009F	gaagggggatcggctagctac	*Sau*3AI
Sgo‐2009R	cactgatcaggtacagaatagccgt	*Sau*3AI
Cloning *S. gordonii pepO* gene		
RW112	tttaagaaggagatatacatatgACACGACTGCAAGATGATTTTTATG	
RW113	cagtggtggtggtggtggtgctcgagCCAAATAATCACACGATCTCC	
	plasmid flanking region in lower case	

### 2.4 Purification of recombinant *S. gordonii* PepO

The *pepO* (SGO_1799) gene was amplified from *S. gordonii* V2016 genomic DNA using primers RW112 and RW113. The fragment was assembled into pET21a, following digest with *Xho*I and *Nde*I, upstream of a 6‐HIS tag using NEB HiFi builder (NEB). The following product was transformed into *E. coli* DH5α cells. Isolated plasmid bearing the insert of interest was verified by transformation into *E. coli* BL21(DE3), induction with 0.5 mmol/L IPTG and the demonstrated ability of protein of the correct molecular mass in the lysate to bind nickel resin. Protein was purified as described in Wilkening, Chang, and Federle (2016). In brief, cells were grown to an OD_600_ of ~0.7 and induced with 0.5 mmol/L IPTG. Cells were grown with agitation at 30°C for 6 hr. Harvested cells were suspended in buffer A (0.2 mol/L phosphate buffer, 0.0054 mol/L potassium chloride, 0.274 mol/L sodium chloride, 20 mmol/L imidazole, 0.07% β‐mercaptoethanol) with the addition of 1x protease inhibitor cocktail (Pierce). Cells were lysed, and the soluble fraction was separated by centrifugation. The resulting lysate was passed over a HisTrap‐HP Nickel column (GE Bioscience) and washed with buffer A. rPepO was then eluted using 10% buffer B (buffer A + 500 mmol/L imidazole). Fractions containing rPepO were assessed by SDS–PAGE, pooled, and concentrated to 2.8 mg/ml via a 50,000 MWCO Amicon column. Imidazole was removed via buffer replacement with buffer C (buffer A with no imidazole). Concentrated rPepO was stored in 20% glycerol at −80°C.

### 2.5 Interaction of peptidases with bacteria and mammalian cells

Because the purified rPepO from bacteria displayed low peptidase activity compared with enzyme associated with bacteria, we studied interaction between the rPepO enzyme and bacteria or mammalian cells, using furin as control. Bacterial cells were those that naturally displayed a low furin‐like peptidase activity, including *S. salivarius* 101‐1, *S. pyogenes* NS05‐24, and *E. coli* JM109 and the *S. gordonii pepO* mutant. *Streptococcus* strains were grown in THY and *E. coli* was in LB broth overnight, and washed in Na/MES before use. Mammalian cells included two cell lines: the human lung carcinoma A549 cells and the human tongue squamous carcinoma SCC9 cells. Cells were grown in the DMEM medium with 2 mmol/L glutamine and 10% fetal bovine serum until confluence. The cells were harvested and washed in Na/MES buffer. One milliliter of washed bacterial cells (about 10^9^ per ml) or mammalian cells (about 10^5^ per ml) were used to perform the assay. Furin‐like peptidase activities of washed cells alone, the cells with added rPepO or Furin proteins, and the purified proteins in buffer without cells as control were assayed for peptidase activity as described above.

### 2.6 Interaction of PepO with bacterial cell wall peptidoglycans

Two bacterial cell wall peptidoglycans were used to interact with furin and the *S. gordonii* PepO protein. One is from *Bacillus subtilis* and the other is from *S. pyogenes*. These two cell walls were tested because *B. subtilis* cell wall was available commercially (Sigma), and *S. pyogenes* cell was isolated in our laboratory for another project. *S. pyogenes* NZ131 was grown overnight in THY broth. Cells were isolated by centrifugation and suspended in 0.1 mol/L Tris–HCl pH 6.8 with 0.25% SDS. The cells in solution were placed inside a conical tube and incubated in a boiling water bath for 2 hr to kill the bacteria and inactivate wall enzymes. Bacterial debris was collected by centrifugation at 5,000 xg in a Beckman JA‐20 rotor (Beckman Coulter) for 10 min. The pellet was washed four times in 0.1 mol/L Tris pH 6.8 to remove residual SDS. The pellet was suspended in 0.1 mol/L Tris (pH 7.5), 200 μg/ml RNase A, 100 μg/ml DNase I, and 0.2 mol/L MgSO_4_ and subjected to mechanical disruption at 15,000 psi using an EmulsiFlex‐C5 homogenizer (Avestin). After disruption, the solution was incubated with shaking at 37°C for 2 hr, allowing for degradation of nucleic acid polymers. Trypsin (250 μg/ml) and 10 mmol/L CaCl_2_ were added to the solution and continue incubation and shaking overnight. Finally, the insoluble fraction was centrifuged and the pellet suspended in 1 N HCl for 4 hr with shaking at 37°C to eliminate associated teichoic acids. Finally, the *S. pyogenes* peptidoglycan was suspended in 1 ml PBS at about 100 mg/ml. The purified recombinant human furin and *S. gordonii* rPepO protein were mixed with these bacterial cell wall peptidoglycans for 60 min at room temperature and assayed for furin activity.

### 2.7 Activation of PepO by proteases

Since interaction with bacteria or cells boosted the furin‐like peptidase activity of the human furin and purified PepO of *S. gordonii*, we tested if other proteases could activate PepO. Briefly, proteases including trypsin, chymotrypsin, papain, pepsin, pronase, protease, and proteinase K (from Sigma) solutions were made at three concentrations, 1 mg/ml, 100 μg/ml, and 10 μg/ml in Na/MES buffer (pH 7.0). The rPepO protein at 2.8 μg was incubated with 100 μl of each enzyme at three concentrations for 30 min at 37°C. Buffer alone and buffer with rPepO without proteases were used as controls. Furin‐like peptidase activities of the protease‐treated rPepO and furin were assayed by the fluorogenic furin substrate Boc‐RVRR‐AMC (Enzo Life Sciences) in the VICTOR X5 Plate Reader of PerkinElmer (Excitation 360 nm; Emission 460 nm).

### 2.8 Bacterial competence assay

*Streptococcus gordonii* V2016 wild‐type and its mutant strains with defect in three peptidases, PepO, PulO and SepM, were grown overnight in THS (TH broth with 5% horse serum) broth. In the second morning, the cultures were diluted 1:40 into fresh THS broth and continued to incubate for 2 hr or at time points indicated in the chart to develop competence. The plasmid pA13 DNA (Em^R^) and a chromosomal DNA that carries an erythromycin resistance marker were added, and the cultures were incubated for 2 hr and plated on TH agar plates containing erythromycin 10 μg/ml. After incubation at 37°C in a candle jar for 24 hr, colonies were counted for transformants.

### 2.9 HPV16 entry assay

293TT cells of an adenovirus transformed human embryonic kidney cell line with a stably integrated SV40 genome with high levels of large T antigen were cultured in DMEM (Dulbecco's Modified Eagle Medium) + Enhanced GLU (GIBCO, Life Technologies, Grand Island, NY) supplemented with 10% fetal bovine serum (Sigma‐Aldrich, St. Louis MO) and 250 ng/ml Hygromycin B (Invitrogen). The HPV16 pseudovirion (PsV) was produced with an Optiprep purification or a maturation method using overnight incubation of crude cell lysate at 37°C. HPV16 PsV packaging plasmids (p16L1‐GFP and pfwB) and the expression vector for luciferase (pCLucf) driven by the CMV promoter were used (Buck & Thompson, 2007). This system relies upon a co‐propagation of L1/L2 expression plasmid together with a reporter plasmid [green fluorescent protein (GFP)] to generate high titers of mature PsV stocks for visualization of viral entry.

PsV particles (20 μl/well) were placed into wells in a 6‐well plate or (5 μl/well) into a 96‐well plate coated with Type I collagen containing 293TT cells at 50%–60% confluence after the furin inhibitor decanoyl‐RVKR‐chloromethylketone (CMK) (ENZO Life Sciences. Farmingdale, NY; stock: 100 mmol/L, working concentration 1.0 μmol/L) was added to suppress internal furin activity of the cell. Infection was monitored by observation under a fluorescent microscope on day 3 and by reading with a fluorometer for GFP expression due to plasmid replication in 293TT cells on day 4 and day 6, post infection.

### 2.10 Statistical analysis

The paired 2‐tailed Student *t* test with a confidence limit of *p *<* *.05 determined the level of significance between two comparative groups, which consisted of control and experimental groups. The results presented in the figures are means of triplicate counts with standard deviations unless otherwise stated.

## 3 RESULTS

### 3.1 Bacterial genes encoding furin‐like peptidase activity

**Figure 1:**
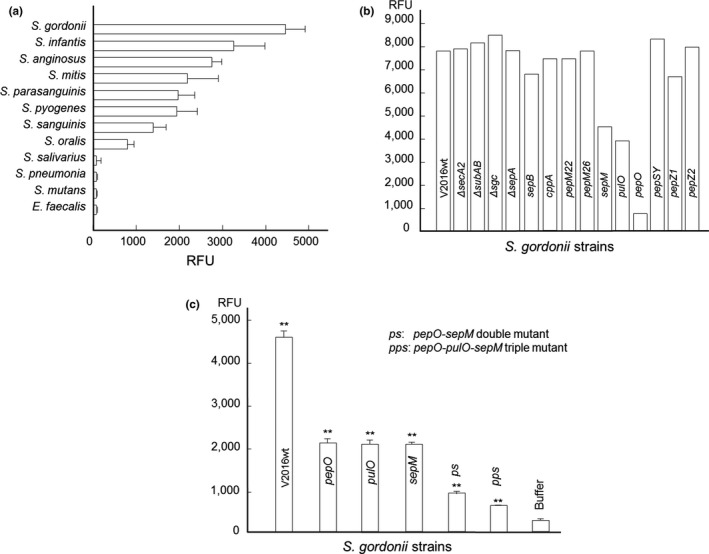
(a) Furin‐like activity of strains representing 12 oral and throat *Streptococcus* and *Enterococcus* species. (b) Furin‐like activities of *S. gordonii* V2016 and its 13 peptidase and the *secA2* mutants (one measure per sample). (c**)** Furin‐like activities of *S. gordonii* and its furin‐like peptidase‐negative mutants. **Statistically very significant difference (*p *<* *.01) between different groups: the wild‐type, single, double and triple mutants, and the buffer control. Each bar represents the mean of triplicate values ± standard deviation

We analyzed 12 oral and throat bacterial strains representing 11 *Streptococcus* and 1 *Enterococcus* species (Table 1) for furin‐like peptidase activities using a fluorogenic furin substrate Boc‐RVRR‐AMC (Enzo Life Sciences) (Cruz, Biryukov, Conway, & Meyers, 2015). Upon incubation of bacterial cells with the substrate, 8 of the 11 streptococcal strains tested were positive for furin‐like activity (73%; Figure 1a), whereas the *Enterococcus* strain was negative. *S. gordonii* V2016 displayed the highest furin‐like peptidase activity. We also tested 19 oral *Lactobacillus* strains for furin‐like activity (Table 1) and found that 18 were negative (no higher than the buffer control), and 1 (*L. rhamnosus* OLB25b) was positive. Thus, in comparison to a panel of *Lactobacillus* strains, the *Streptococcus* species were more likely to express extracellular furin‐like enzymes.

To identify genes encoding furin‐like peptidases in *Streptococcus*, we used the human furin protein sequence to BLAST against the genomic sequences of the *Streptococcus* genus. The subtilisin‐like serine protease was found as a homolog. However, blasting the *S. gordonii* genome (Vickerman, Iobst, Jesionowski, & Gill, 2007) with the furin sequence did not yield significant similarity. We then performed in silico analysis of *S. gordonii* genome for all genes encoding putative peptidases and identified 47 genes. As furin is an endopeptidase, we dismissed genes encoding exopeptidases (aminopeptidases and carboxypeptidases), leaving 14 genes to consider as candidates for furin activity. Among them, two genes, SGO_0316 and SGO_0317, are linked, and thus, a total of 13 genetic loci were subjected to mutagenesis with the kanamycin resistance marker to achieve allelic exchange (Pavlova et al., 2013) or inactivation by insertion duplication with the plasmid pSF151 (Tao, 1998) (Table 1). In addition, to identify if the Sec2 system of *S. gordonii* (Bensing & Sullam, 2009) is required for secretion of these furin‐like peptidases without a typical signal peptide, we constructed a *secA2* deletion mutant.

Each mutant was subjected to the furin‐like activity assay with the fluorogenic furin substrate. Results showed that inactivation of 3 genetic loci, SGO_1799, SGO_0664 and SGO_0652, each had reduced furin‐like activity (Figure 1b). We used chromosomal DNA isolated from one mutant as a substrate for natural transformation by another mutant containing a different antibiotic resistance marker to generate double‐ and triple‐protease mutants (Tao, 1998). These mutants showed further reduced furin‐like activity compared with the wild‐type and single‐gene knockout mutants (Figure 1c), suggesting that these enzymes have redundant activities. Among the three proteins, none contain a classical signal peptide for secretion, and since the *secA2* mutation (Bensing & Sullam, 2009) did not affect furin‐like activity in *S. gordonii* V2016, alternative pathways unrelated to Sec or SecA2 may allow for the observed extracellular peptidase activity.

The SGO_1799 locus encodes a 630‐amino acid protein with a calculated molecular weight of 71.5 kDa and is homologous to the conserved endopeptidase O (PepO) in other bacteria (Oetjen, Fives‐Taylor, & Froeliger, 2001). Without a signal peptide or a transmembrane domain, *S. gordonii* PepO is presumably a cytoplasmic protein and/or an extracellular protein secreted by an alternative pathway. Due to a zinc‐binding site, it is a presumed zinc‐dependent metalloendopeptidase. Separated by 148 and 149 base pairs from its neighboring genes, *S. gordonii pepO* appears to be an independent gene, not part of a multicistronic operon.

The SGO_0664 locus encodes a 220‐amino acid endopeptidase with a calculated molecular weight of 24.7 kDa. The protein does not have a signal peptide but has 4‐6 transmembrane domains. It is predicted that the protein may be an intramembrane peptidase specifically cleaving transmembrane domains of other integral membrane proteins. It is homologous to the prepilin signal peptidase PulO of other bacteria. Separated by 169 base pairs, *S. gordonii pulO* is divergently located upstream to the gene encoding Dps (DNA starvation/stationary phase protection protein), but overlaps convergently by 31 base pairs with the tryptophan biosynthesis (*trp*) operon downstream. The *pulO* gene also appears to be independent and not part of an operon.

The SGO_0652 locus encodes a 347‐amino acid with a calculated molecular weight of 38.1 kDa. Without a signal peptide, this locus is homologous to the conserved *sepM* gene in *S. mutans*, which encodes a membrane‐associated peptidase required for processing competence stimulating peptide (CSP) for competence development and quorum sensing (Biswas, Cao, Kim, & Biswas, 2015; Hossain & Biswas, 2012). With one transmembrane domain, *S. gordonii sepM* is also predicted as a membrane‐associated peptidase. The genomic location shows that *S. gordonii sepM* appears to be the last gene in an operon with two other genes, which encode phosphopantetheine adenylyltransferase (*coaD*) involved in coenzyme A biosynthesis and rRNA methyltransferase. It is unknown why the three seemingly unrelated genes co‐transcribe in an operon.

To test if these genetic loci in other *Streptococcus* species also encode peptidases with furin‐like activity, we transformed chromosomal DNA isolated from *S. gordonii pepO, pulO*, and *sepM* mutants into *S. sanguinis* SK36. We were able to obtain transformants with *S. gordonii pepO* and *pulO* DNAs, but not with *sepM* DNA (Table 1). Furin assay with these two *S. sanguinis* peptidase mutants showed reduction of furin‐like peptidase activity in comparison with the wild‐type strain SK36. Therefore, at least two furin‐like endopeptidases, PepO and PulO, may be present in multiple oral streptococcal species.

### 3.2 Interacting with cells and chymotrypsin activates *S. gordonii* rPepO

**Figure 2:**
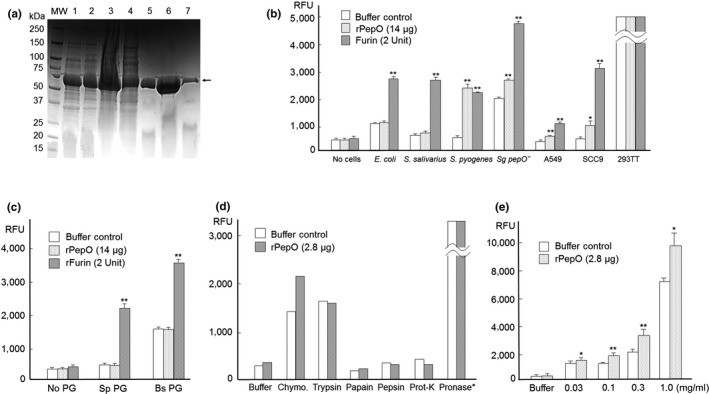
(a) Affinity purification of recombinant *S. gordonii* PepO from *E. coli* shown in SDS–PAGE. 1) Pellet without IPTG; 2) Pellet with IPTG for 6 hr; 3) Crude lysate; 4) Elution fraction 2 (flow through); 5) Fraction 8; 6) Fraction 9; and 7) Fraction 10. Arrow: rPepO. (b) rPepO activation by bacteria (*E. coli* JM109, *S. salivarius* 101‐1, *S. pyogenes* NS05‐24, and *S. gordonii pepO* mutant) and mammalian cells (A549, SCC9 and 293TT). Buffer controls are buffer with indicated cells. 293TT cells released about 19,000 RFU fluorescence from the furin substrate with or without added rPepO and furin, showing high endogenous furin level. (c) rPepO activation by bacterial peptidoglycan (PG). Sp. *S. pyogenes*; Bs, *B. subtilis*. Buffer controls are buffer with indicated PG. Note: *B. subtilis* PG in buffer displayed high furin‐like protease activity, indicating that these PGs may contain proteases. (d) rPepO activation by chymotrypsin, trypsin, papain, pepsin, and proteinase K. Pronase (protease from *Streptomyces griseus*) (Calbiochem) released 12,000 RFU fluorescence from the furin substrate with or without added rPepO (one measure per sample). Buffer controls are buffer with indicated protease. (e) rPepO activation by chymotrypsin. Both buffer control and rPepO samples contain chymotrypsin with indicated amount in the figure. *Significant difference (*p *<* *.05); **very significant difference (*p *<* *.01) compared with controls

We cloned and expressed the *S. gordonii pepO* gene tagged with 6xHIS in *E. coli* and purified the recombinant protein (rPepO) using a HisTrap‐HP Nickel column (GE Bioscience), and SDS–PAGE analysis confirmed its molecular size and purity (Figure 2a). The rPepO protein was concentrated to about 2.8 mg/ml and tested for furin‐like peptidase activity, but its peptidase activity was no higher than a buffer control. We then tested the possibility that activity of rPepO, or the human furin protein (rFurin, New England BioLabs), would be enhanced in the context of bacterial or mammalian cells. The *S. gordonii pepO* knockout mutant and other cells, including *S. salivarius* 101‐1, *S. pyogenes* NS05‐24, and *E. coli* JM109 bacterial strains, and the mammalian SCC9 and A549 cells, all naturally expressing furin‐like peptidase activity, were combined with purified rPepO and added to the fluorogenic substrate. Results (Figure 2b) showed that all six cells activated rFurin, but only four (except *E. coli* and *S. salivarius*) activated rPepO. Data from 293TT could not be interpreted due to high intrinsic furin‐like activity of the cell. In addition, bacterial peptidoglycan preparations from *Streptococcus pyogenes* and *Bacillus subtilis* both activated rFurin, but not rPepO (Figure 2c), indicating that rPepO may require activation that is more specific. By interacting with various proteases, including trypsin, chymotrypsin, papain, pepsin, pronase, protease, and proteinase K, only chymotrypsin promoted furin‐like activity of rPepO (Figure 2d) and in a dose‐dependent manner (Figure 2e). As pronase (protease from *Streptomyces griseus*) (Calbiochem) showed strong furin‐like peptidase activities with and without added rPepO on the fluorogenic furin substrate, it could not be used for evaluating rPepO activation.

### 3.3 PepO inactivation reduces competence in *S. gordonii*

**Figure 3:**
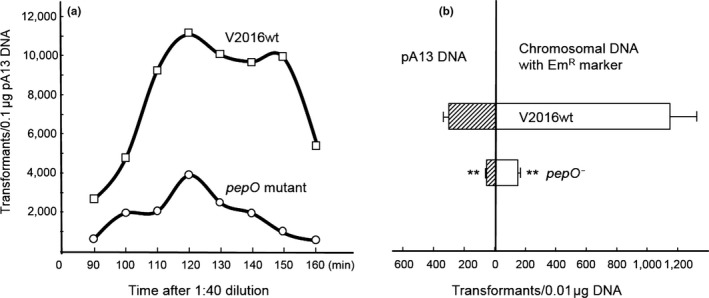
Transformation of *S. gordonii* V2016 and its *pepO*‐defective mutant. (a) Competence development as function of time. (b) Transformation rates between plasmid and chromosomal DNAs. Comparing with the wild type, the *pepO* mutant shows significant decrease in both plasmid and chromosomal DNA transformations. **Very significant difference (*p *<* *.01) when compared with the control

While generating the double and triple mutants, we noticed reduction of transformation efficiency with chromosomal DNA in these *S. gordonii* peptidase‐defective mutants. Therefore, we studied their genetic transformation. As shown in Figure 3a, the peak number of transformants was reached at 120 min after a 1:40 dilution for both the wild‐type V2016 and the *pepO* mutant, but the *pepO* mutant displayed substantially reduced transformation rates. When compared among three different peptidase‐defective mutants against the wild type for uptake of circular plasmid and linear chromosomal DNAs, the *pepO* mutant showed the highest reductions in transformation by both DNA types (Figure 3b), while the *pulO* and *sepM* mutants only showed moderate reductions in transformation by chromosomal DNA (data not shown). Both *pepO* and *sepM* mutants had a same growth rate as the wild‐type strain V2016 with a doubling time of 41 min, while the *pulO* mutant had a slower growth rate with a doubling time of 63 min. Therefore, inactivation of *pepO* might suppress competence development of *S. gordonii* independent of growth rate. However, addition of purified PepO protein to the competent *S. gordonii pepO* mutant culture (at 8 μg/ml) prior to the addition of plasmid or chromosomal DNA did not restore its rate of transformation to the level of the wild‐type strain (data not shown).

### 3.4 *S. gordonii* PepO promotes HPV infection

**Figure 4:**
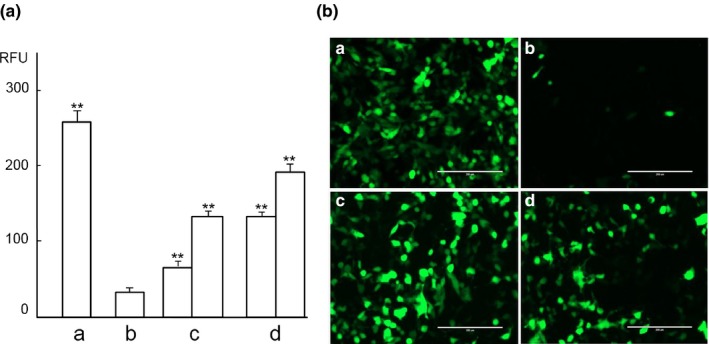
HPV16 PsV infection of 293TT cells boosted by *S. gordonii* rPepO. (A) HPV16 PsV infection on day 6 detected by fluorometer. a, HPV16 PsV. b, HPV‐16 PsV + CMK. c, HPV‐16 PsV + CMK + furin (2 Units, left; 10 Units, right). d, HPV‐16 PsV + CMK + *S. gordonii* rPepO (14 μg, left; 56 μg, right). **Very significant difference (*p *<* *.01) when compared with the control (b). (B) HPV16 PsV infection on day 3 detected by fluorescent microscope. Bar: 200 μm. a, HPV16 PsV infection of 293TT. B, HPV‐16 PsV infection of 293TT inhibited by CMK. c, HPV‐16 PsV infection of 293TT with CMK promoted with 10 units of furin. d, HPV‐16 PsV infection of 293TT with CMK promoted with 56 μg of rPepO

We studied HPV16 pseudovirus (PsV) entry into the 293TT cells to evaluate the role of furin‐like activity of *S. gordonii* PepO in viral activation. Since the 293TT cell has endogenous furin activity, to study the role of exogenous furin‐like peptidase, we added the furin inhibitor decanoyl‐RVKR‐chloromethylketone (CMK) to suppress its endogenous furin activity (Richards et al., 2006). As shown in Figure 4Aa and Ba, without CMK treatment, the HPV16 PsV displayed high rates of infection into 293TT cells promoted by its endogenous furin. With CMK treatment (Figures 4Ab and Bb), HPV16 PsV entry into 293TT cells was significantly reduced due to inhibition of endogenous furin. Addition of exogenous furin (Figures 4Ac and Bc) or the *S. gordonii* PepO protein (Figures 4Ad and Bd) to these cells, in which the endogenous furin was suppressed by CMK, HPV16 PsV entry into 293TT cells showed significant increase (*p *<* *.01) in a dose‐dependent manner in comparison with the CMK‐treated control cells (Figures 4Ab and 4Bb). The *S. gordonii PepO* protein‐treated group (Figure 4Ad) showed a higher HPV16 PsV entry than the human furin‐treated one (Figure 4Ac).

## 4 DISCUSSION

It has been reported that bacteria colonizing the human airway activate influenza viruses by cleaving the viral surface glycoprotein hemagglutinin with peptidase (Böttcher‐Friebertshäuser, Klenk, & Garten, 2013). However, there have been no reports on HPV activation by bacteria. Unlike influenza viruses, activation of HPV requires the cleavage of its L2 protein at the furin consensus site (‐RXXR↓‐) (Richards et al., 2006; Schiller et al., 2010). To our knowledge, this is the first report of a furin‐like peptidase in bacteria capable of promoting HPV16 infection in vitro.

After screening 12 representative strains of *Streptococcus* and *Enterococcus* species for furin‐like peptidases, we found that *S. gordonii* V2016 displayed the highest furin‐like peptidase activity among tested strains. *S. gordonii* not only colonizes the human oral cavity but also the throat (Frandsen, Pedrazzoli, & Kilian, 1991), which is a hot spot for HPV infection‐associated cancers. By analyzing the *S. gordonii* genome, we identified 14 genes encoding endoproteases and constructed null mutations in these genes. Defects in any of the three genes, *pepO, pulO,* and *sepM*, showed reduction in furin‐like peptidase activity (Figure 1B). Although the initial screening showed that the *pepO* mutant had the most reduction, repeated assays with triplicate samples showed comparable reductions in furin‐like peptidase among these three mutants (Figure 1C).

PepO, a member of the M13 family of zinc metalloendopeptidases, has been well studied in several different bacteria. *Streptococcus parasanguinis* PepO is similar to the human endothelin‐converting enzyme 1 (ECE‐1) (Oetjen et al., 2001). In *Streptococcus pneumoniae* (Agarwal et al., 2013, 2014), *S. pyogenes* (Honda‐Ogawa et al., 2017), *Streptococcus mutans* (Alves et al., 2017) and *P. gingivalis* (Ansai, Yu, Urnowey, Barik, & Takehara, 2003), PepO is a virulence protein facilitating evasion of innate immunity and invasion of host cells. In *S. pyogenes*, PepO degrades peptide pheromones of a quorum sensing system (Wilkening et al., 2016).

PulO is homologous to the prepilin peptidase (PilD) involved in Type IV pili production in other bacteria. These pili were found recently in the Gram‐positive bacterium *Streptococcus sanguinis* as a part of filamentous nano‐machine for bacterial motility (Gurung et al., 2016). In *S. pneumoniae*, the type IV pilus mediates DNA binding during natural transformation (Laurenceau et al., 2013). The *S. gordonii* CSP induces PulO expression (Vickerman et al., 2007). We found that inactivation of *pulO* retarded *S. gordonii* growth rate by about 50%.

SepM, a cell membrane‐associated peptidase, is required for the CSP maturation in *S. mutans* (Biswas et al., 2015; Hossain & Biswas, 2012). SepM proteins from *S. mutans, S. pyogenes* and *S. agalactiae* all activate the *S. mutans* CSP by cleaving CSP‐21 between the Ala18 and Leu19 residues, but SepM from *S. gordonii* does not (Biswas et al., 2015).

Although these peptidases exit in many bacteria, their furin‐like activity may be species specific. By transforming chromosomal DNAs from *pepO* and *pulO*‐inactivated *S. gordonii* strains into *S. sanguinis* SK36, the furin‐like activity reduced in the latter (data not shown). This suggests that PepO and PulO in *S. sanguinis* may have furin‐like activity, similar to that of *S. gordonii*. However, these peptidases may not have or have weak furin‐like activities in *Streptococcus salivarius, S. pneumoniae, S. mutans*, and *Enterococcus faecalis*, because the tested strains representing these species did not show detectable furin‐like activities (Figure 1a).

Among these three furin‐like peptidases, PulO and SepM are membrane proteins that are bound to the bacterial cell surface. This may limit their ability to interact with viruses. PepO is not membrane‐bound, so it may be released into the environment to interact with viruses in distance. Therefore, we focused our efforts on characterizing *S. gordonii* PepO.

After cloning and purification of *S. gordonii* PepO, we found it had a low activity against the fluorogenic furin‐specific substrate. Apparently, protein maturation may be required. By interacting with different bacteria, cells, bacterial peptidoglycans, and multiple different proteases, we found that chymotrypsin enhanced *S. gordonii* PepO in a dose‐dependent manner (Figure 2e). This suggests that maturation of *S. gordonii* PepO may require a chymotrypsin‐like protease. Interestingly, interaction with two bacterial peptidoglycans increased the activity of rFurin (Figure 2c). The mechanism is unknown, but it may be due to bacterial peptidoglycan‐associated protease. For example, the *B. subtilis* peptidoglycan with buffer alone displayed high furin‐like activity.

Without a signal peptide, PepO normally would be considered as a cytoplasmic protein. However, because inactivation of *pepO* substantially reduced the furin‐like peptidase activity, PepO appears to be present outside the cell. Since furin‐like peptidase activity is not reduced in the *secA2* mutant, a Sec or SecA2 independent pathway (Bensing & Sullam, 2009) might secrete PepO. In *Streptococcus pneumoniae* (Agarwal et al., 2013), *S. pyogenes* (Honda‐Ogawa et al., 2017) and *Porphyromonas gingivalis* (Ansai et al., 2003), PepO is secreted by an unknown pathway to facilitate bacteria invasion into host cells.

Because inactivation of *pepO* substantially reduced competence in *S. gordonii*, the intended function of PepO might be associated with competence development. Although *S. gordonii pepO* expression is not induced by CSP (Vickerman et al., 2007), at least two peptidases in *Streptococcus*, ComA (Ishii et al., 2010) and SepM (Biswas et al., 2015), have been reported to promote competence by processing CSP to its mature form. The mechanism by which *S. gordonii* PepO facilitates genetic transformation is unknown, but it appears to play this role intracellularly, because adding purified PepO to competent *pepO* mutant culture did not restore its transformation to the level of the wild type. This is different from the SepM peptidase in *S. mutans*, which works extracellularly to process CSP (Hossain & Biswas, 2012). Nonetheless, linking PepO to bacterial competence is novel, as currently identified bacterial competence factors have not included PepO (Straume, Stamsås, & Håvarstein, 2015). Although PepO was identified to inactivate CSP in *S. pneumoniae*, it was not proven to regular competence in this bacterium (Bergé, Langen, Claverys, & Martin, 2002).

The proprotein convertase furin not only is essential for activation of certain viruses such as HPV (Richards et al., 2006; Schiller et al., 2010) but also promotes cancer development and metastasis (Jaaks & Bernasconi, 2017). HPV‐induced cancers mostly occur in the cervix and oropharynx. Unlike the cervix, where furin expression is high and exists in all layers of the vaginal epithelium (Kines et al., 2009), furin expression in the oral epithelium is low, only detectable in the upper spinous and surface layers, but not in the lower basal layer, where HPV initiates infection (López de Cicco et al., 2002). We observed that *S. gordonii* PepO promoted HPV16 PsV infection of cultured 293TT cells in vitro in a dose‐dependent manner similar to that of furin. This suggests that bacterial furin‐like peptidases might compensate the deficiency of host furin in oral epithelium basal cells to assist HPV infection. Therefore, *Streptococcus* PepO, perhaps also PulO and SepM, might have a moonlighting role (Henderson, 2014) in activation of viruses, such as HPV, as a furin‐like peptidase.

The human saliva contains small amounts of proteases including furin and furin inhibitors of the host origin (Sun, Salih, Oppenheim, & Helmerhorst, 2009). The trace amount of furin in the saliva may promote oral HPV infection and tumorigenesis, but due to presence of nearly equal amount of furin inhibitors, such as alpha‐1‐antitrypsin, in the saliva, the effect of salivary furin, if any, might be minimal. Therefore, furin‐like peptidases released by oral bacteria might play a role in oral HPV infection and tumorigenesis. The microbiome findings have been varied. One group reported decrease (Schmidt et al., 2014), while two other groups reported increase (Guerrero‐Preston et al., 2016; Mukherjee et al., 2017) of *Streptococcus* species in oral cancer lesions in comparison with controls. We think that microbial cancer‐promoting factors might not be limited to specific bacterial species. Furin‐like peptidases released by any microorganism could promote oral cancer. Identifying *Streptococcus* peptidases with furin‐like activity that promotes HPV infection may help us understand the linkage among *Streptococcus* colonization sites, HPV tissue tropism, and HPV‐associated tumorigenesis. Because only some, not all, bacterial species studied showed a high furin‐like activity, identification of specific furin‐positive bacteria and/or bacteria‐released furin‐like peptidases may provide novel drug targets for blocking viral infection and subsequent carcinogenesis.

About 18% of the global cancer burden has been attributed to infectious agents, including hepatitis B and C viruses, HPV, EBV, and *Helicobacter pylori* (Vedham, Divi, Starks, & Verma, 2014). However, most infected individuals do not develop cancer. Pathogenic infections are necessary but are not sufficient for cancer initiation. Additional cofactors, including secondary infections by different microbial agents, may be required. Our study, for the first time, has revealed the possibility that bacterial furin‐like peptidases might promote viral infection. Further studies will be needed to characterize such bacterium–virus interaction in vivo.

## CONFLICT OF INTEREST

None declared.
